# Valine Treatment Enhances Antimicrobial Component Production in Mammary Epithelial Cells and the Milk of Lactating Goats Without Influencing the Tight Junction Barrier

**DOI:** 10.1007/s10911-023-09529-x

**Published:** 2023-02-18

**Authors:** Yusaku Tsugami, Takahiro Nii, Naoki Isobe

**Affiliations:** Graduate School of Integrated Sciences for Life, Hiroshima University, 1-4-4 Kagamiyama Higashi-Hiroshima, 739-8528 Hiroshima, Japan

**Keywords:** Antimicrobial component, Branched-chain amino acid, Mammary gland, Tight junction, Valine

## Abstract

The production of antimicrobial components and the formation of less-permeable tight junctions (TJs) are important in the defense system of lactating mammary glands and for safe dairy production. Valine is a branched-chain amino acid that is actively consumed in the mammary glands and promotes the production of major milk components like β-casein; additionally, branched-chain amino acids stimulate antimicrobial component production in the intestines. Therefore, we hypothesized that valine strengthens the mammary gland defense system without influencing milk production. We investigated the effects of valine in vitro using cultured mammary epithelial cells (MECs) and in vivo using the mammary glands of lactating Tokara goats. Valine treatment at 4 mM increased the secretion of S100A7 and lactoferrin as well as the intracellular concentration of β-defensin 1 and cathelicidin 7 in cultured MECs. In addition, an intravenous injection of valine increased S100A7 levels in the milk of Tokara goats without influencing milk yield and milk components (i.e., fat, protein, lactose, and solids). In contrast, valine treatment did not affect TJ barrier function either in vitro or in vivo. These findings indicate that valine enhances antimicrobial component production without influencing milk production and TJ barrier function in lactating mammary glands; thus, valine contributes to safe dairy production.

**Supplementary Information:**

The online version contains supplementary material available at 10.1007/s10911-023-09529-x.

## Introduction

Mastitis is the most frequent disease of the mammary glands and causes substantial economic losses in the dairy industry [1]. Mastitis is caused by pathogens such as *Escherichia coli* and *Staphylococcus aureus*, which invade through the teat canals [2, 3]. To manage the safety and stability of dairy production, methods for preventing mastitis must be established. During lactation, the mammary glands employ a defense system for protection against mastitis-causing pathogens, part of which are the production of antimicrobial components and the formation of less-permeable tight junction (TJs) [4, 5].

In the mammary glands, various antimicrobial components are produced and secreted into milk [4]. The epithelial cells of the mammary glands produce β-defensin 1, lactoferrin, S100A7, and cathelicidin 7 [6, 7], which show nonspecific antibacterial activity against many pathogens, including *E. coli* and *S. aureus*. Defensins and cathelicidins can destroy microbial membranes via cationic charges [8], lactoferrin exhibits bacteriostatic effects through its biding capacity with iron, which is required for the growth of pathogens [9], and S100A7 has antimicrobial activity against *E. coli* [10]. Therefore, the enhanced levels of these antimicrobial components in milk help prevent the growth of pathogens and mastitis.

TJs are formed between mammary epithelial cells (MECs) and composed of claudins and occludin. TJs regulate the movement of water, ions, and small molecules through paracellular pathways [11]. A claudin subtype at the TJ region determines permeability [12]. In the mammary glands during lactation, claudin 3 is localized at TJs in a concentrated manner and contributes to lowering permeability [13], thereby preventing mixing of blood components into milk and pathogens invading the host body [5]. Therefore, improving TJ barrier function will contribute to the safe and stable management of dairy production.

In the present study, we investigated the ability of valine to regulate antimicrobial component production and TJ barrier function in the mammary glands. Valine is a branched-chain amino acid; branched-chain amino acids account for approximately 40% of amino acids absorbed into the mammary glands [14]. Valine promotes the production of β-casein and α-lactalbumin in cultured porcine MECs [15]. β-casein is an abundant protein in milk, and α-lactalbumin is an enzyme involved in the synthesis of lactose. Valine also promotes milk fat production in cultured porcine MECs [16]. Furthermore, branched-chain amino acids are known to stimulate β-defensin production in the intestines of pigs [17]. Moreover, valine supplementation has been shown to improve intestinal barrier function by regulating the mRNA expression of TJ protein in fishes [18, 19]. Therefore, we hypothesized that valine strengthens antimicrobial component production and/or TJ barrier function without influencing milk production in MECs and the mammary glands. We tested this hypothesis using cultured MECs and the mammary glands of lactating Tokara goats.

## Materials and Methods

### Animals

Five healthy Tokara goats (milk yield: 70–295 mL/udder/day; weight: 25.6–33.8 kg; age: 2–5 years, parity: 1–2) at the mid-lactation stage and unafflicted by mastitis (no clinical symptoms) were used in the in vivo experiment, in which they were intravenously injected with valine. Goats were individually housed under a 14:10 h light:dark cycle, fed 0.6 kg hay cubes and 0.2 kg barley each day, and had free access to water and trace-mineralized salt blocks. The feed was offered twice daily at 08:00 and 15:00 h, and the feed supply (energy, protein, and minerals) was calculated based on the Japanese feeding standard for sheep (Ministry of Agriculture, Forestry and Fisheries in Japan, 1996) as reported previously [20]. The goats were milked manually once daily at 08:00 h. Prior to injections, valine (#V0014, Tokyo Chemical Industry, Tokyo, Japan) was dissolved in distilled water at 80 mg/mL, and 6 mL of this solution was intravenously injected at days 0–2 after milking. All experiments were approved by the Animal Research Committee of Hiroshima University (no. C21-20) and conducted according to the Guidelines for Animal Experiments prescribed by Hiroshima University.

### Cell Culture

We established an MEC culture model based on a previously described model [21, 22]. Goat MECs (GMECs) were cultured in 24-well plates or 12-well cell culture inserts (0.4 μm pore size; BD Biosciences, Bedford, MA, USA) coated with collagen gel (Cellmatrix Type 1-A; Nitta Gelatin, Osaka, Japan) with a growth medium [Dulbecco’s modified Eagle medium/Ham’s F-12 (DMEM/F12) medium supplemented with 5% fetal bovine serum, 5 µg/mL ITS-X (1 mg/mL insulin, 0.55 mg/mL transferrin, 0.67 mg/mL selenium, and 0.20 mg/mL ethanolamine; Wako, Osaka, Japan), 10 ng/mL epidermal growth factor (BD Biosciences), and 5 mM sodium acetate (Nacalai Tesque, Kyoto, Japan)] for 6 days until confluence. Subsequently, the GMECs were cultured in a differentiation medium [1% fetal bovine serum, 5 µg/mL ITS-X, 1 ng/mL epidermal growth factor, 5 mM sodium acetate, 1 µg/mL prolactin (provided by A. F. Parlow; lot AFP7170E; NHPP, NIDDK, Torrance, CA, USA), and 1 µM dexamethasone (Sigma-Aldrich, St. Louis, MO, USA) in DMEM/F12 medium]. The upper chamber of the insert was filled with Hanks’ balanced salt solution (HBSS; Thermo Fisher Scientific, Waltham, MA, USA) for differentiation. After 2 days of culture, the GMECs were treated with valine (#V0014; Tokyo Chemical Industry), which was first dissolved in DMEM/F12 medium at 50 mg/mL and then adjusted to concentrations of 1, 2, and 4 mM using normal DMEM/F12 medium.

The epithelial barrier was evaluated in terms of its transepithelial electrical resistance (TEER) and the flux of fluorescein through the barrier, following methods reported in previous studies [23, 24]. To measure the TEER, the electrodes of a Millicell-ERS-2 Voltohmmeter (Millipore, Billerica, MA, USA) were placed in the upper and lower chambers of the insert, and the resistance was measured. To measure fluorescein isothiocyanate (FITC) permeability, the upper chamber of the insert was filled with HBSS containing 10 µg/mL fluorescein sodium salt (molecular weight: 376; Sigma-Aldrich), whereas the lower chamber was filled with the medium alone. The medium from the lower chamber was collected 3 h after the addition of fluorescein, and its paracellular flux was measured using a fluorometer (excitation: 492 nm; emission: 520 nm).

### Analysis of Milk and Blood Samples

Collected milk samples were centrifuged at 1,000 × *g* and 4 °C for 10 min. Milk fat and skim milk were separated from the somatic cell pellets. The cell pellets were either resuspended in phosphate-buffered saline (PBS) to determine their somatic cell count (SCC), which was measured with a Countess II FL Automated Cell Counter (Thermo Fisher Scientific), or in Sepasol-RNA I Super G (Nacalai Tesque) to extract total RNA. The skim milk was stored at − 30 °C for later use in an enzyme-linked immunosorbent assay (ELISA). The Na^+^ level was measured using a LAQUAtwin Na-11 pocket meter (HORIBA, Kyoto, Japan). Milk components (fat, protein, lactose, and solids) were measured using a LactoScope Filter (Delta Instruments LLC, Northvale, NJ, USA).

Blood was collected from the jugular vein and transferred to a tube coated with the anticoagulant heparin. Blood samples were then centrifuged at 1,700 × *g* and 4 °C for 15 min. The leukocyte pellets were washed with distilled water three times and resuspended in Sepasol-RNA I Super G to extract total RNA. Free amino acids in plasma were analyzed using an automatic amino acid analyzer (JLC 500; JEOL, Tokyo, Japan) as reported [25].

### Immunofluorescence

Immunofluorescence analyses were performed using the reported methods [26, 27]. The mammary gland tissues were fixed, dehydrated, and embedded in paraffin. Sections of the tissues (3 μm thick) were then air-dried on MAS-coated slides. After deparaffinization, antigen retrieval was performed by autoclaving the sections in a citric acid buffer (pH 6.0) for 20 min at 121 °C. Cultured GMECs on collagen gel isolated from the insert were fixed with methanol for 10 min at − 20 °C and then with 1% paraformaldehyde in PBS for 10 min at 4 °C. Subsequently, the tissue sections and cells were washed with PBS for 10 min, after which they were incubated in PBS-T (PBS containing 0.05% Tween-20) containing 5% bovine serum albumin (MP Biomedicals) for 1.5 h at room temperature. The sections and cells were then incubated overnight at 4 °C with rabbit polyclonal antibodies against L-amino acid transporter (LAT)-1 (#ab208776; Abcam, Cambridge, UK, 1:100), LAT3 (#ab254719; Abcam, 1:100), and claudin 3 (#34-1700, Thermo Fisher Scientific, 1:200) or with mouse monoclonal antibodies against occludin (#sc-133,256; Santa Cruz Biotechnology, 1:200) diluted in PBS-T containing 2.5% bovine serum albumin. To evaluate immunofluorescence, the sections and cells were incubated with secondary antibodies (Alexa Fluor 488–conjugated goat anti-rabbit, #A32731, 1:400; Alexa Fluor 555–conjugated goat anti-mouse, #A32727, 1:400; both Thermo Fisher Scientific) diluted with PBS-T containing 2.5% bovine serum albumin for 1 h at room temperature. Immunofluorescence images were obtained using a fluorescence microscope (BZ-9000) and processed using analysis software (Keyence, Osaka, Japan).

### Reverse Transcription Polymerase Chain Reaction

Reverse transcription polymerase chain reaction (RT-PCR) was performed according to the reported methods [28]. Total RNA from normal mammary gland tissues, cultured GMECs, somatic cells in milk, and leukocytes in blood was extracted using Sepasol-RNA I Super G. Reverse transcription was performed using ReverTra Ace qPCR RT Master Mix (Toyobo, Osaka, Japan), and RT-PCR was conducted using an Applied Biosystems™ 2720 Thermal Cycler (Thermo Fisher Scientific) with Brilliant III Ultra-Fast SYBR QRTPCR (Agilent Technologies, Santa Clara, CA, USA). The cycling conditions were 95 °C for 5 min, followed by 40 cycles of 95 °C for 15 s, 60 °C for 30 s, and 72 °C for 30 s. The primers used are listed in Suppl. Table 1. Finally, the samples were separated on 2.5% agarose gels and stained with EtBr solution (Bio-Rad Laboratories, Hercules, CA, USA).

#### ELISA

To measure the levels of β-defensin 1, lactoferrin, S100A7, and cathelicidin 7, a competitive ELISA was performed as reported in previous studies [6, 7, 29].

A sandwich ELISA was performed to measure the levels of albumin and IgG using an anti-albumin antibody (Life Laboratory Company, Yamagata, Japan, 1:5,000), goat-albumin antibody-horseradish peroxidase (HRP) (#A50-103P; Bethyl Laboratories, Montgomery, TX, USA, 1:30,000), goat-IgG antibody (#A50-104 A; Bethyl Laboratories, 1:3,000), and goat-IgG antibody-HRP (#A50-104P; Bethyl Laboratories, 1:30,000).

### Western Blotting

Western blotting was performed using a reported method [26, 30]. The lysates were lysed in a Laemmli sodium dodecyl sulfate–solubilizing buffer and heated for 15 min at 70 °C. The samples were then separated on sodium dodecyl sulfate–polyacrylamide gels and transferred to polyvinylidene difluoride membranes (Bio-Rad Laboratories). Immunosignals were detected using claudin 3 (#34-1700, 1:1,000), claudin 4 (#PA5-32354, Thermo Fisher Scientific, 1:1,000), α-tubulin (#GTX628802; GeneTex, Los Angeles, CA, USA, 1:10,000), secondary HRP–conjugated anti-rabbit antibody (Abcam, 1:5,000), anti-mouse antibody (Sigma-Aldrich, 1:5,000), and Immobilon Forte Western HRP Substrate (Millipore). Images of the bands were obtained using Ez-Capture II (Atto). The bands were quantified using a CS Analyzer 4.0 (Atto).

### Statistical Analysis

Data are expressed as means ± standard deviation (SD). Statistical analyses were performed using SAS software (version 9.4; SAS Institute Inc.). Significant differences at *p* < 0.05 were determined using one-way ANOVA with a post-hoc Tukey’s test (Fig. 2), Student’s t-test (Figs. 3 and 4E), or Dunnett’s test (Figs. 4B and D and 5, and 6). The in vivo experiments were performed using ten udders of five different goats, whereas the in vitro experiments were performed using three different GMECs derived from different goats. In the in vitro experiment, the number of samples was defined as n = 1 for a sample derived from a well.

**Fig. 1:**
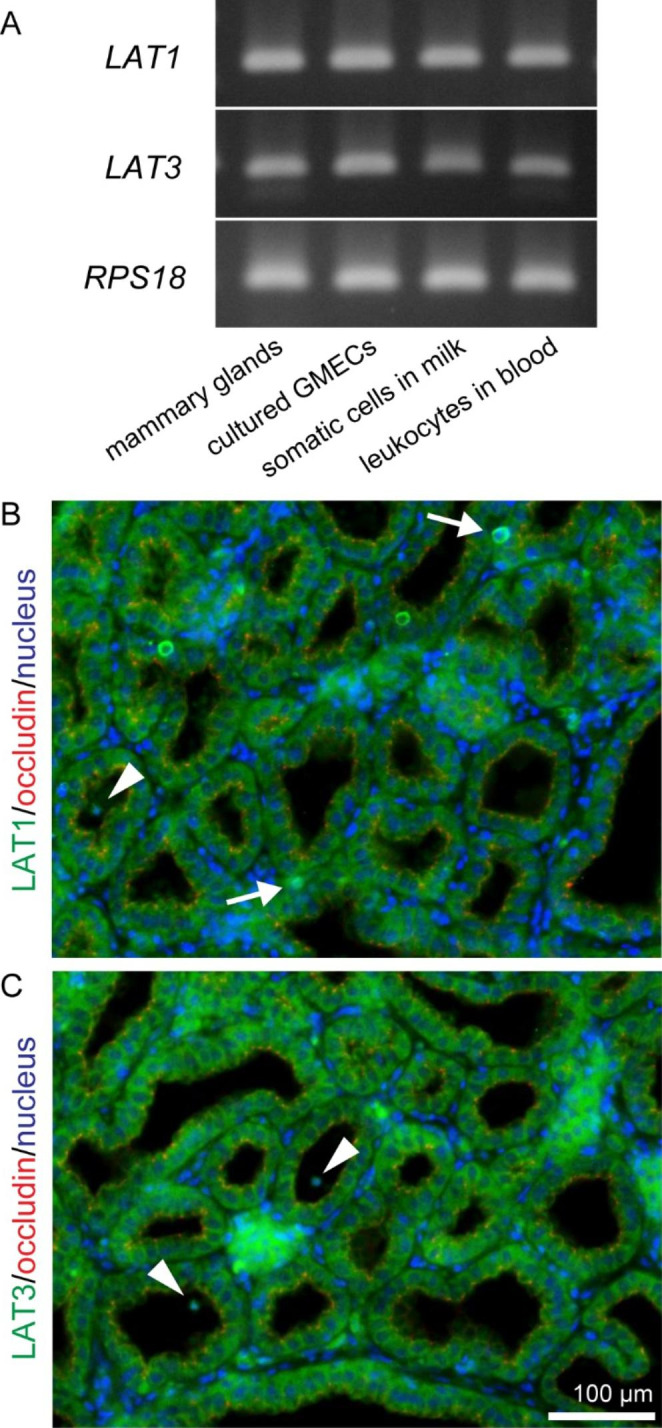
mRNA expression and localization of LAT1 and LAT3 in goat mammary glands. (A) Bands show the results of RT-PCR for *LAT1*, *LAT3*, and *RPS18* in mammary glands, cultured goat mammary epithelial cells (GMECs), somatic cells in milk, and leukocytes in the blood of Tokara goats. Immunofluorescence images show the localization of LAT1 (green) (B) and LAT3 (green) (C) in mammary gland tissue. Occludin (red) was used as a marker of most apical regions in lateral membranes. Single cells in the mammary stromal regions (arrows) and the mammary alveolus (arrowheads) showed positive reactions against LAT1 or LAT3 antibodies. Scale bar, 100 μm

**Fig. 2:**
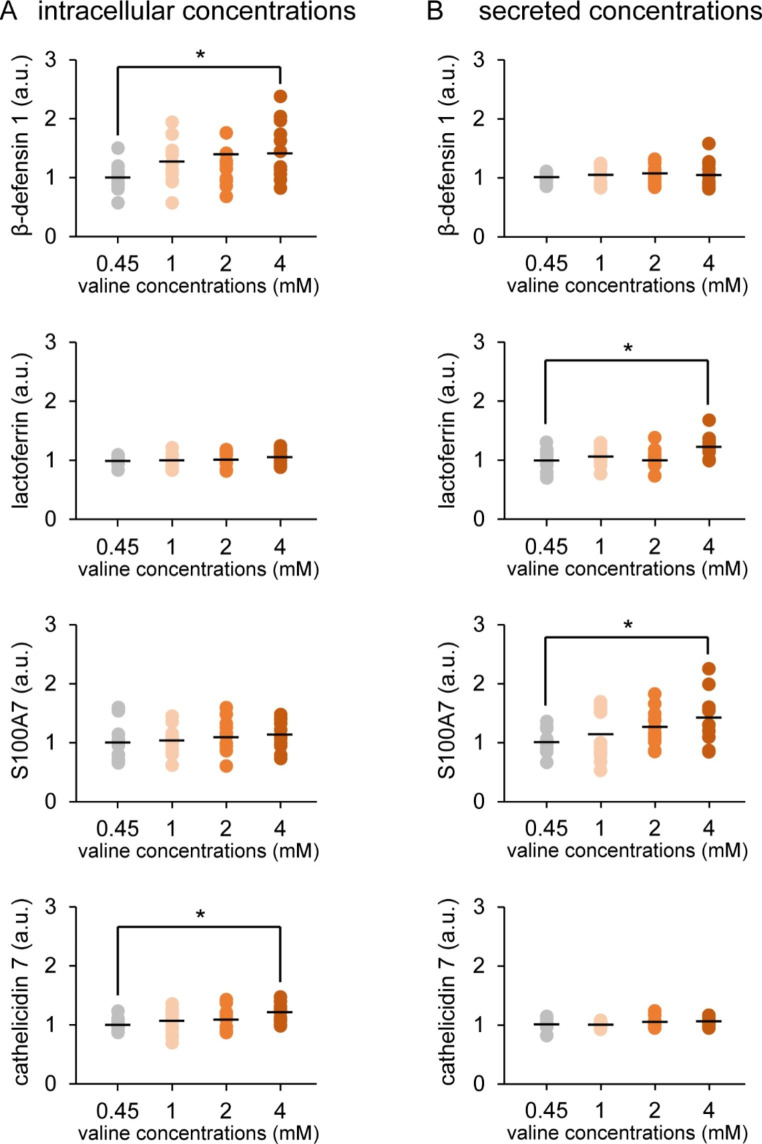
Influence of valine on antimicrobial component production in cultured goat mammary epithelial cells (GMECs). Graphs show the intracellular concentrations (A) and secreted concentrations into the medium (B) of β-defensin 1, lactoferrin, S100A7, and cathelicidin 7. Antimicrobial component concentrations were detected using ELISA in GMECs treated with valine at the given concentrations. Control GMECs were affected by valine at 0.45 mM. Asterisks show significant differences (*p* < 0.05) between groups according to Tukey’s test (n = 12)

**Fig. 3:**
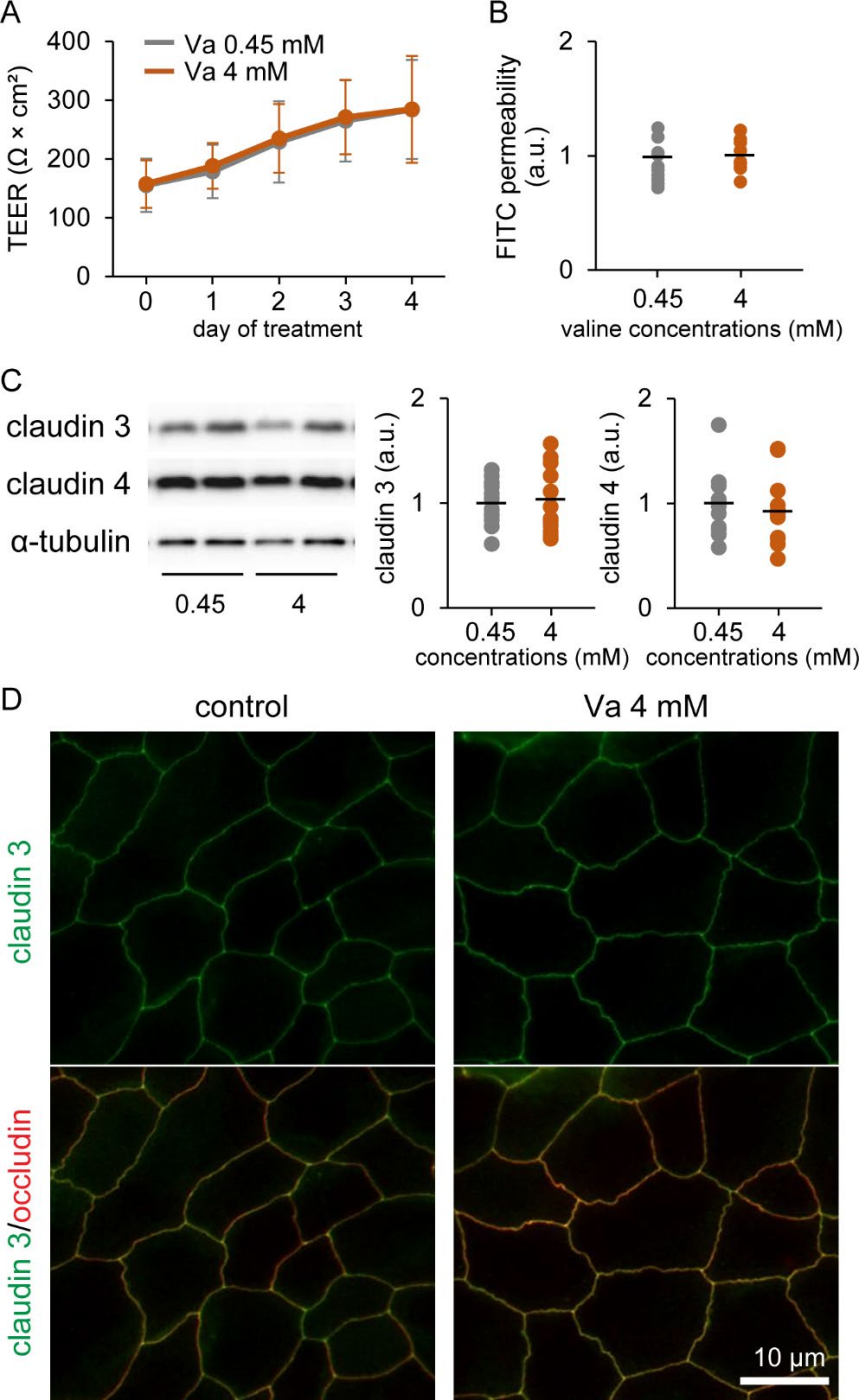
Influence of valine on tight junction (TJ) barrier function in cultured goat mammary epithelial cells (GMECs). Graphs show the transepithelial electrical resistance (TEER) (A) and fluorescein isothiocyanate (FITC) permeability (B) in GMECs treated with 4 mM valine for 4 days (n = 10–12). (C) Western blotting of claudin 3 and claudin 4 in GMECs treated with valine at 4 mM for 3 days. Graphs show the results of densitometry analysis, with α-tubulin used as an internal control (n = 12). (D) Localization of claudin 3 (green) in GMECs treated valine at 4 mM for 4 days. Occludin (red) was used as the TJ marker. Scale bar, 10 μm

**Fig. 4:**
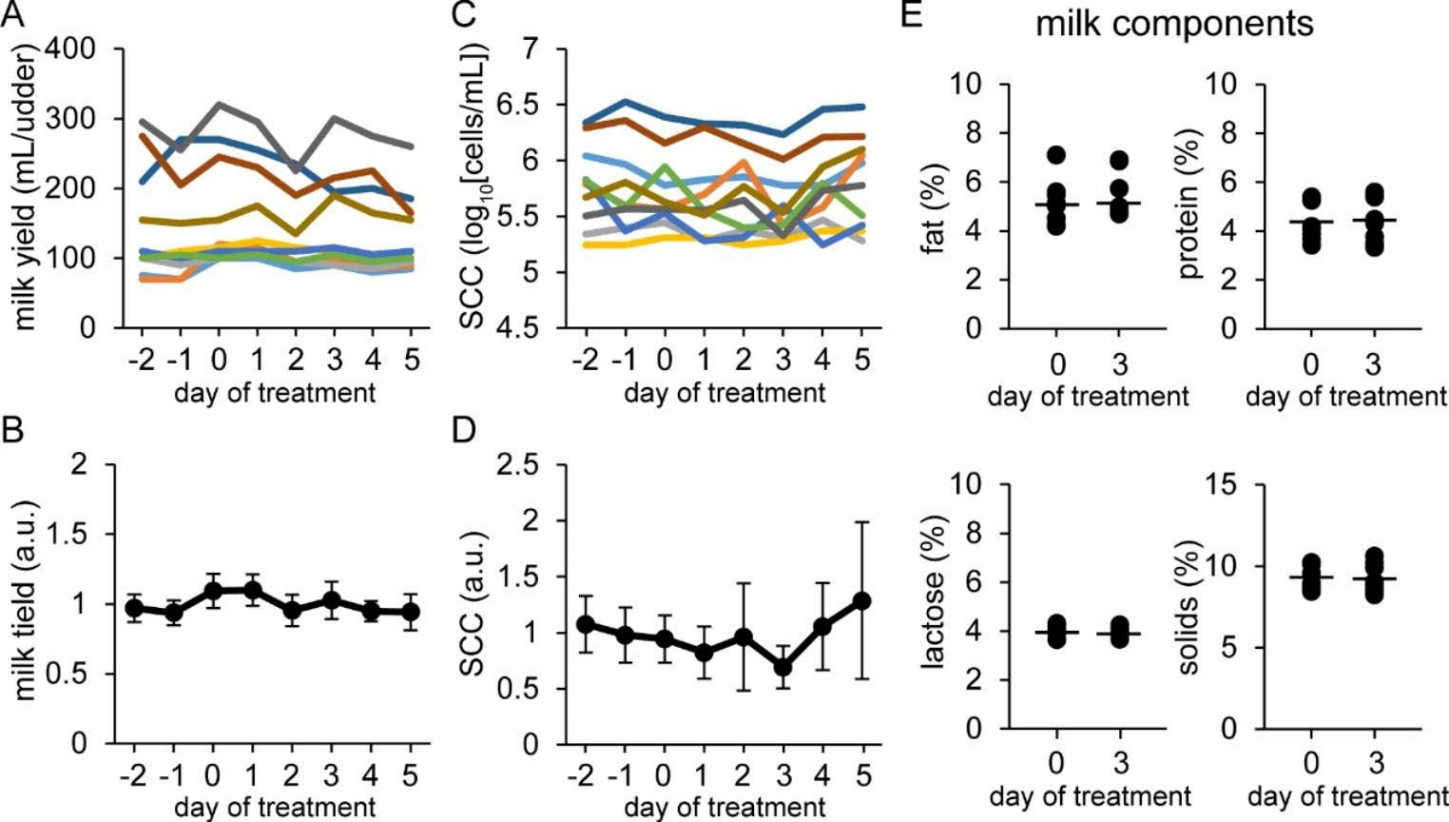
Influence of intravenous injections of valine on milk yield, somatic cell count (SCC) in milk and milk components, and the tight junction barrier in mammary glands Tokara goats were intravenously injected with 6 mL of valine at 80 mg/mL on days 0–2. Upper graphs show changes in milk yield (A) and SCC (C) in individual udders, whereas lower graphs (B and D) show changes in the relative values of these variables compared with the average of pretreatment (days − 2 to 0), i.e., the control. Colored lines show the changes in milk yield and SCC derived from the same udder. (E) Milk components (fat, protein, lactose, and solids) on days 0 and 3. Data are means ± SD (n = 10)

**Fig. 5:**
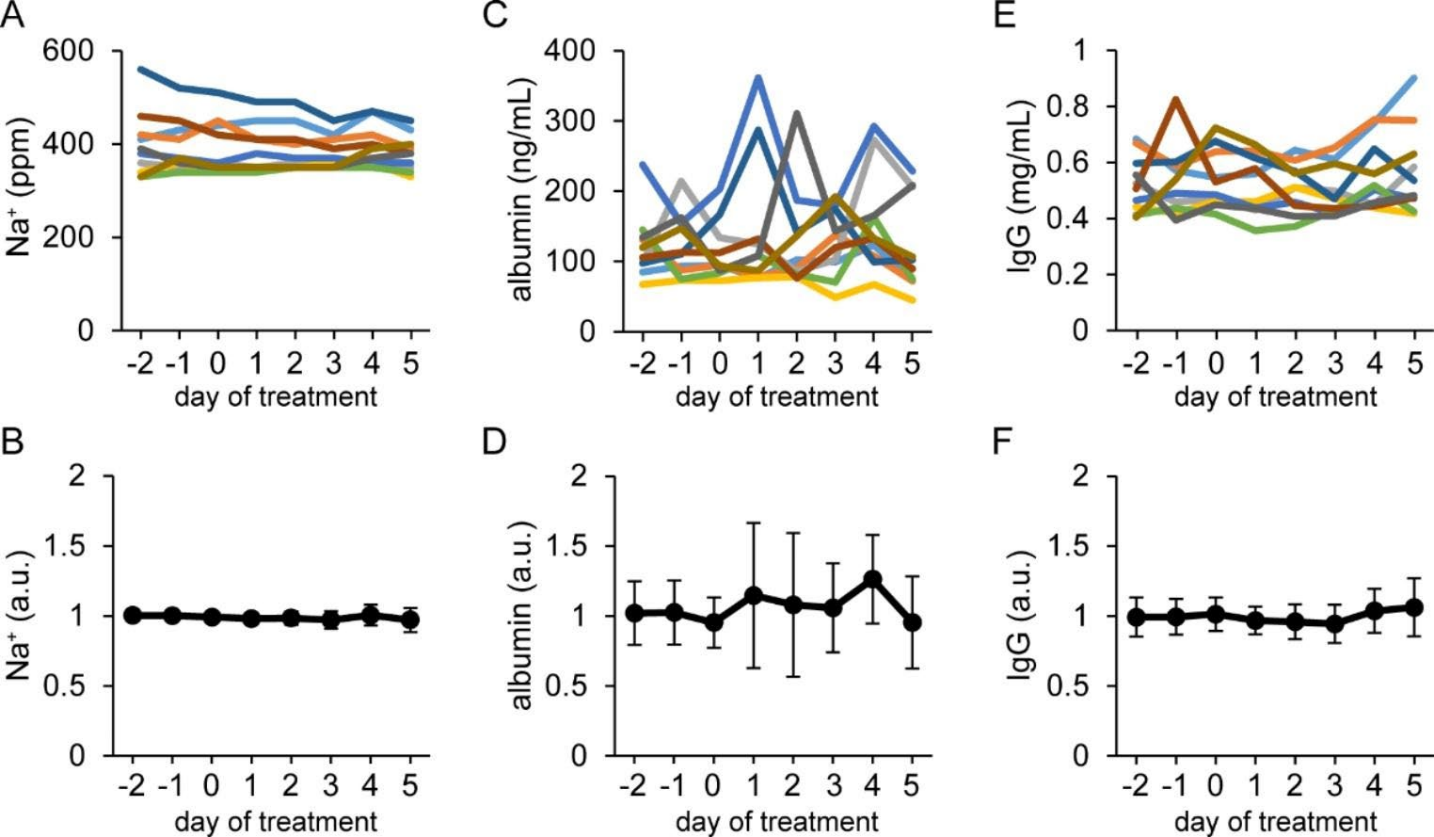
Influence of intravenous injections of valine on the blood-derived components in milk Tokara goats were intravenously injected with 6 mL of valine at 80 mg/mL on days 0–2. Upper graphs show changes in Na^+^ level (A), albumin level (C), and IgG level (E) in milk of individual udders, whereas lower graphs (B, D, and F) show changes in the relative values of these variables compared with the average of pretreatment (days − 2 to 0), i.e., the control. Colored lines show the changes in blood-derived components in milk derived from the same udder. Data are means ± SD (n = 10)

**Fig. 6:**
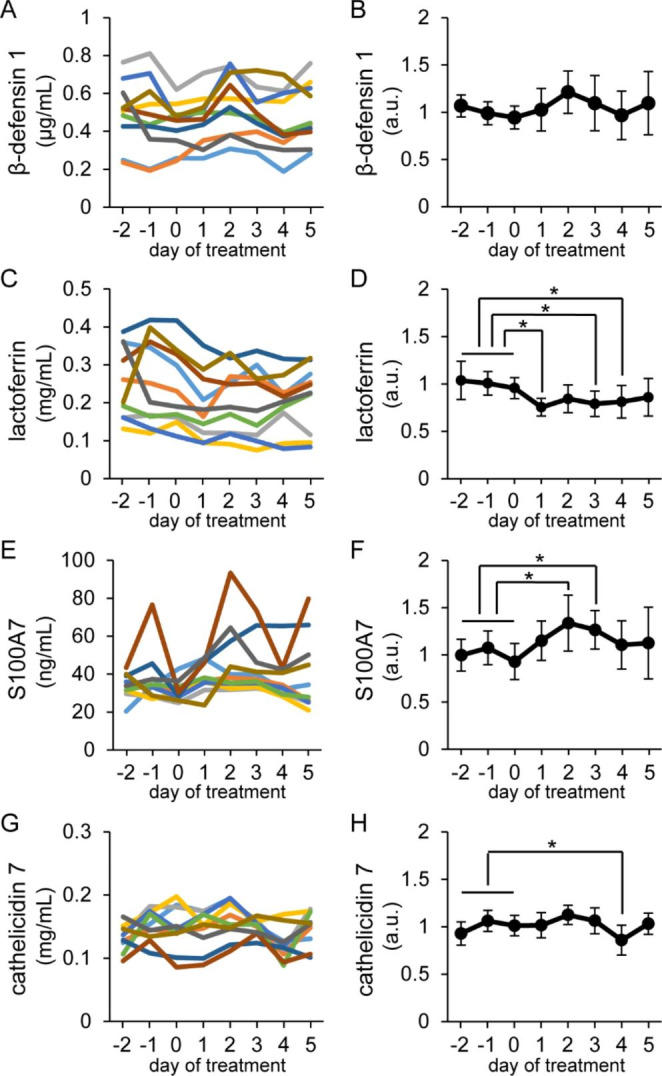
Influence of intravenous injections of valine on antimicrobial component concentrations in milk Tokara goats were intravenously injected with 6 mL of valine at 80 mg/mL on days 0–2. Left graphs show changes in β-defensin 1 (A), lactoferrin (C), S100A7 (E), and cathelicidin 7 (G) levels in individual udders, whereas right graphs (B, D, F, and H) show changes in the relative values of these variables compared with the average of pretreatment (days − 2 to 0), i.e., the control. Colored lines show the changes in antimicrobial components in milk derived from the same udder. Data are means ± SD (n = 10). Asterisks indicate significant differences (*p* < 0.05 versus the control) according to Dunnett’s test

## Results

### mRNA Expression and Localization of LAT1 and LAT3 in Goat Mammary Glands

According to our RT-PCR results (Fig. 1A), the bands of LAT1 (SLC7A5; 123 bp) and LAT3 (SLC7A7; 112 bp) representing mRNA expression were detected in all samples of mammary glands, cultured GMECs, somatic cells in goat milk, and leukocytes in blood. In the mammary glands, LAT1 and LAT3 were localized in the basolateral membranes of epithelial cells (Fig. 1B and C). In addition, single cells in the mammary alveolus or in the stromal region showed positive reactions against LAT1 and LAT3.

### Effects of Valine on Antimicrobial Component Production in Cultured GMECs

According to our ELISA results (Fig. 2), cultured GMECs treated with valine at 4 mM for 3 days exhibited significantly increased intracellular concentrations of the antimicrobial components β-defensin 1 (1.4-fold increase) and cathelicidin 7 (1.2-fold increase) compared with those of the control group (valine at 0.45 mM) (Fig. 2A). In addition, to determine the effect of valine on the secretion of antimicrobial components, GMECs were treated with 4 mM valine for 4 days, considering the time-lag between the synthesis of milk components in the cells and their subsequent secretion. Compared with the control group, valine treatment significantly increased the secretion of lactoferrin (1.2-fold increase) and S100A7 (1.4-fold increase) into the medium in the upper chamber (Fig. 2B).

### Effects of Valine on TJ Barrier Function in Cultured GMECs

We determined the influence of valine on TJ barrier function in GMECs by measuring TEER and FITC permeability, finding that valine treatment at 4 mM for 4 days did not affect TEER and FITC permeability (Fig. 3A and B). According to our western blotting results (Fig. 3C), the levels of claudin 3 and claudin 4 in GMECs were not affected by valine treatment at 4 mM for 3 days. In addition, using immunofluorescence, we observed the localization of claudin 3 in cultured GMECs (Fig. 3D). In control GMECs, claudin 3 was colocalized with the TJ marker occludin, and the valine-treated GMECs exhibited the same localization of claudin 3 observed in the control group.

### Influence of Valine Injection into the Blood on Milk Production and TJ Barrier Function

In lactating goats, we confirmed that an intravenous injection of valine affected plasma concentrations of valine, which were significantly increased 15 min after the injection (0 min: 177.2 ± 11.0 µM; 15 min: 426.5 ± 45.8 µM [*p* < 0.001]). Valine was intravenously injected daily for 3 days, but the plasma concentration of valine barely changed in this period (day 0: 159.2 ± 45.8 µM; day 1: 164.7 ± 42.0 µM; day 2: 164.6 ± 47.2 µM; day 3: 171.8 ± 52.3 µM; day 5: 174.6 ± 55.0 µM).

Milk yield, SCC, and milk components (fat, protein, lactose, and solids) were not altered by valine injection (Fig. 4). In addition, the levels of the blood-derived components (Na^+^, albumin, and IgG) in milk were not changed by valine treatment (Fig. 5).

### Effect of Valine Injection into the Blood on Antimicrobial Component Production in the Mammary Glands

According to our ELISA results (Fig. 6), the S100A7 levels in milk were increased at days 2–3 after valine treatment by more than 1.2-fold those of the control group (average of pretreatment: days − 2 to 0). In contrast, the levels of lactoferrin and cathelicidin 7 decreased during or immediately after valine treatment.

## Discussion

In the present study, valine treatment enhanced S100A7 secretion in both cultured GMECs and lactating mammary glands. S100A7 shows antimicrobial activity against *E. coli* [10]; therefore, treatment could contribute to preventing *E. coli*-induced mastitis. In the current study, valine treatment also increased intracellular concentrations of β-defensin 1 and cathelicidin 7 in cultured GMECs. Some pathogens such as *S. aureus* parasitize MECs and adversely affect milk production; however, increases in antimicrobial component concentrations in MECs can repress such parasitism [31]. Therefore, valine may also contribute to preventing *S. aureus*-induced mastitis. Overall, our study indicates that valine enhances the production of some important antimicrobial components in lactating mammary glands.

Mammary glands affected by mastitis or in the postweaning stage exhibit disrupted TJ barrier function, which increases SCC or blood-derived components in milk [32, 33]. In the present study, valine treatment did not affect TJ barrier function in either lactating mammary glands or cultured GMECs, and the level and localization of claudins were the same in the control and valine treatment groups. Claudin 3 is a major TJ protein in the mammary glands during lactation, whereas claudin 4 levels increase when inflammation increases [23, 34]. Our findings indicate that valine barely affects the TJ barrier function of lactating mammary glands. An amino acid–based oral rehydration solution containing valine has been reported to repress the disruption of intestinal TJ barrier function caused by total body irradiation [35]. This suggests that valine might improve damage to TJ barrier function, which occurs in mammary glands affected by mastitis. In our previous study, loose TJs in the mammary glands induced high antimicrobial component production by increasing the frequency of antigen exposure [26]. In contrast, valine treatment enhanced antimicrobial component production without affecting TJ barrier function.

In the present study, valine treatment enhanced the secretion of lactoferrin in cultured GMECs, whereas valine injection reduced lactoferrin levels in milk. Lactoferrin is produced by both MECs and leukocytes [22]. In the mammary glands, LAT1 and LAT3 act as transporters of valine [36, 37], and these proteins were expressed in both MECs and leukocytes. Further studies are needed to determine the influence of valine on leukocytes. We found that valine plasma concentrations were normal after 24 h, even when injections were repeated three times; however, the S100A7 and lactoferrin levels in milk were altered by valine injections. Thus, valine was apparently rapidly consumed, including in MECs and leukocytes. Further studies are required to determine whether supplementation of rumen-protected valine also regulates antimicrobial component production.

## Conclusion

In this study, we investigated the effects of valine on the antimicrobial component production and TJ barrier function of lactating mammary glands. Valine enhanced the production of some antimicrobial components, but did not affect milk production and TJ barrier function. Therefore, valine treatment could contribute to stable and safe dairy production and the prevention of mastitis via antimicrobial component production.

## Electronic Supplementary Material

Below is the link to the electronic supplementary material.

Supplementary Material 1

## Data Availability

The datasets generated and analyzed during the current study are available from the corresponding author on reasonable request.
