# Correction to: Valine Treatment Enhances Antimicrobial Component Production in Mammary Epithelial Cells and the Milk of Lactating Goats Without Influencing the Tight Junction Barrier

**DOI:** 10.1007/s10911-023-09533-1

**Published:** 2023-03-14

**Authors:** Yusaku Tsugami, Takahiro Nii, Naoki Isobe

**Affiliations:** Graduate School of Integrated Sciences for Life, Hiroshima University, 1-4-4 Kagamiyama Higashi- Hiroshima, 739-8528 Hiroshima, Japan

**Correction to: Journal of Mammary Gland Biology and Neoplasia 28, 3 (2023)**

10.1007/s10911-023-09529-x

Following the publication of the original article [1], it was noted that due to renumbering of figure citations in the text, the figure images and captions were paired incorrectly. Figure citations, images and captions were amended accordingly.

The original article has been corrected.
