# Supplementary material for: Valine Treatment Enhances Antimicrobial Component Production in Mammary Epithelial Cells and the Milk of Lactating Goats Without Influencing the Tight Junction Barrier
Source: J Mammary Gland Biol Neoplasia. 2023 Feb 18;28(1):3. doi: 10.1007/s10911-023-09529-x (PMC9938821; doi:10.1007/s10911-023-09529-x)
Supplement: Supplementary file 1 — Supplementary Material 1 [file 10911_2023_9529_MOESM1_ESM.docx]

Suppl. Table 1: Primer sequences used for RT-PCR

| Gene | Accession  number | Primer | Product  size |
| --- | --- | --- | --- |
| *LAT1*  *(SLC7A5)* | XM_005691915.3 | (F) ACGGCTGCTGACGCCTGTAC | 123 |
|  |  | (R) GCCACGCAGAGCCAGTTGAAG |  |
| *LAT3*  *(SLC7A7)* | XM_005691915.3 | (F) GCATCGCTTGCCAGGATGTC | 112 |
|  |  | (R) ATCTGCCCCTCTCCATTGGC |  |
| *RPS18* | NM_001285639.1 | (F) TAATCCCGCCGAACCCCATT | 125 |
|  |  | (R) GGTGTGTACAAAGGGCAGG |  |
